# 

**Published:** 1919-12

**Authors:** 


					^7
DECEMBER, 1919.
Vol. XXXVI. No. 137.
THE
BRISTOL
MEDICOCHIRU RGICAL
AL
PUBLISHED UXDEIt THE AUSPICES OF r>
THE BRISTOL MEDICO -CHI RURG 1CAJ&S0C IETY.
Editor: P. WATSON-WILLIAVMS
,F6W
WITH WHOM ARK ASSOCIATE
J. LACY FIRTH, M.S., M.D., JAMES SWAIN
CAREY COOMBS, M.D.
J. A. NIXON, C.M.G., M.D., Assistant Editor.
Editorial Secretary: J. M. FORTESCUE-BRICKDALE, M.A., M.D.
BRISTOL: J. W. ARROWSMITH LTD.
London : Simpkin, Marshall, Hamilton, Kent & Co. Ltd.
Price One Shilling and Sixpence net.

				

